# Decoding Size Distribution Patterns in Marine and Transitional Water Phytoplankton: From Community to Species Level

**DOI:** 10.1371/journal.pone.0127193

**Published:** 2015-05-14

**Authors:** Leonilde Roselli, Alberto Basset

**Affiliations:** Department of Biological and Environmental Sciences & Technologies, University of Salento, Lecce, Italy; Texas A&M University at Galveston, UNITED STATES

## Abstract

Understanding the mechanisms of phytoplankton community assembly is a fundamental issue of aquatic ecology. Here, we use field data from transitional (e.g. coastal lagoons) and coastal water environments to decode patterns of phytoplankton size distribution into organization and adaptive mechanisms. Transitional waters are characterized by higher resource availability and shallower well-mixed water column than coastal marine environments. Differences in physico-chemical regime between the two environments have been hypothesized to exert contrasting selective pressures on phytoplankton cell morphology (size and shape). We tested the hypothesis focusing on resource availability (nutrients and light) and mixed layer depth as ecological axes that define ecological niches of phytoplankton. We report fundamental differences in size distributions of marine and freshwater diatoms, with transitional water phytoplankton significantly smaller and with higher surface to volume ratio than marine species. Here, we hypothesize that mixing condition affecting size-dependent sinking may drive phytoplankton size and shape distributions. The interplay between shallow mixed layer depth and frequent and complete mixing of transitional waters may likely increase the competitive advantage of small phytoplankton limiting large cell fitness. The nutrient regime appears to explain the size distribution within both marine and transitional water environments, while it seem does not explain the pattern observed across the two environments. In addition, difference in light availability across the two environments appear do not explain the occurrence of asymmetric size distribution at each hierarchical level. We hypothesize that such competitive equilibria and adaptive strategies in resource exploitation may drive by organism’s behavior which exploring patch resources in transitional and marine phytoplankton communities.

## Introduction

The underlying mechanisms driving species coexistence have long been the subject of intense debate [1, 2]. Fifty years after Hutchinson’s “paradox of the plankton” [3], interspecific coexistence in phytoplankton is still a major issue in community ecology. Several coexistence mechanisms, accounting for niche partitioning [4], such as environmental fluctuation [3, 5, 6], temporal succession [7], trophic [8] or chaotic population dynamics [9] have been proposed to explain species diversity. These niche-based mechanisms can indeed promote coexistence and shape community size structure [10].

Body size is a trait of individuals that affects organism’s physiology and ecology [11] and commonly differentiates coexisting species as an indirect niche dimension [12]. Phytoplankton cell size and shape (hereafter regarded as surface to volume ratio) are morphological traits directly related to the fitness of the individuals since they affects growth, metabolism and access to resources [6, 13]. Trait-based approaches are increasingly used in phytoplankton ecology to explain and predict community organization along environmental gradients [14, 15]. Quantitative relationships have been observed with key individual processes such as nutrient and light uptake [16, 14], intra-cellular nutrient transport rates [17], sinking behaviour [18] and anti-predator strategies [19,20]. On the other hand, individual cell size and size distributions are known to be affected by physical factors, including water temperature [21, 22], turbulence [7, 23, 24, 25] and mixed layer depth [26, 27], adding niche dimensions as selective forces to the process of phytoplankton cell size optimisation. Marine and freshwater scientists have addressed the adaptive advantages of being of the optimal size [7, 13, 28, 29]. Theoretically, small cells should dominate low-nutrient environments while large cells should have a competitive advantages in nutrient-rich conditions [30, 31]. However, the competitive advantage of small phytoplankton taxa over larger ones has been observed in a wide range of conditions including nutrient limitation [28, 32] and enrichment [33–38], light limitation and shading [16, 39, 40], decrease in mixing intensity [25, 41] and shallow mixed layer depth [26]. From theoretical perspective to fossil evidence phytoplankton is supposed to evolve toward small size [28, 42, 43], nevertheless, large cells have acquired a wide range of adaptive strategies that compensate for the competitive disadvantages arising from their larger size. Larger cells have nutrient storage capacity in nutrient-rich and fluctuating environments, [5, 26, 44], motility and ability to control buoyancy in high physical mixing conditions [45], lower metabolic costs in fluctuating or high irradiance conditions [29, 40, 46, 47], alternative metabolic pathways [48], anti-predator strategies against grazing pressure [49]. Recently, Litchman et al. [26] have addressed morpho-functional adaptations in diatoms from an evolutionary perspective, comparing selective pressures on phytoplankton cell size of freshwater and marine environments. They showed smaller diatoms maybe selected for phosphorous-limited and shallow mixed layer depth freshwaters than larger cells in marine environments. A combination of nutrient storage capability and sinking behaviour of cells in response to different nutrient regime and mixed layer depth has been proposed to explain these patterns [26, 27].

Here, we follow a similar approach, by comparing transitional water and coastal marine environments and addressing the whole phytoplankton community with quantitative field data, extending Litchman et al. [26] hypotheses on the selective mechanisms the phytoplankton cell size distribution. Transitional waters (e.g. shallow coastal lagoons) are functional ecotones physically connected with coastal waters through tidal channels, characterised by shallow well-mixed column waters and typically richer in nutrients than marine environments [50, 51]. We address several questions. Do high-resource conditions confer a competitive advantage select for large cells over small cells or on the contrary, small cells are favoured under rich nutrient conditions? What niche dimensions are important for understanding mechanisms of phytoplankton community assembly? Do phytoplankton respond to a specific niche dimension adopting a specific size or a specific functional behaviour? We test the hypothesis that several niche dimensions may exert contrasting selective pressures on phytoplankton cell size. In order to address this hypotheses we 1) compare patterns in functional and size structure in transitional and coastal waters; 2) analyse the drivers of these patterns, if any, by describing the relationship between phytoplankton traits, resource availability and physical properties of the water column; 3) investigate potential mechanisms driving cell morphology adaptation.

## Material and Methods

No specific permissions were required for these locations/activities because the areas for sampling were specifically included in the projects so the authorities require no specific permission. We confirm that the field studies did not involve endangered or protected species.

### Study sites

**Fig 1:**
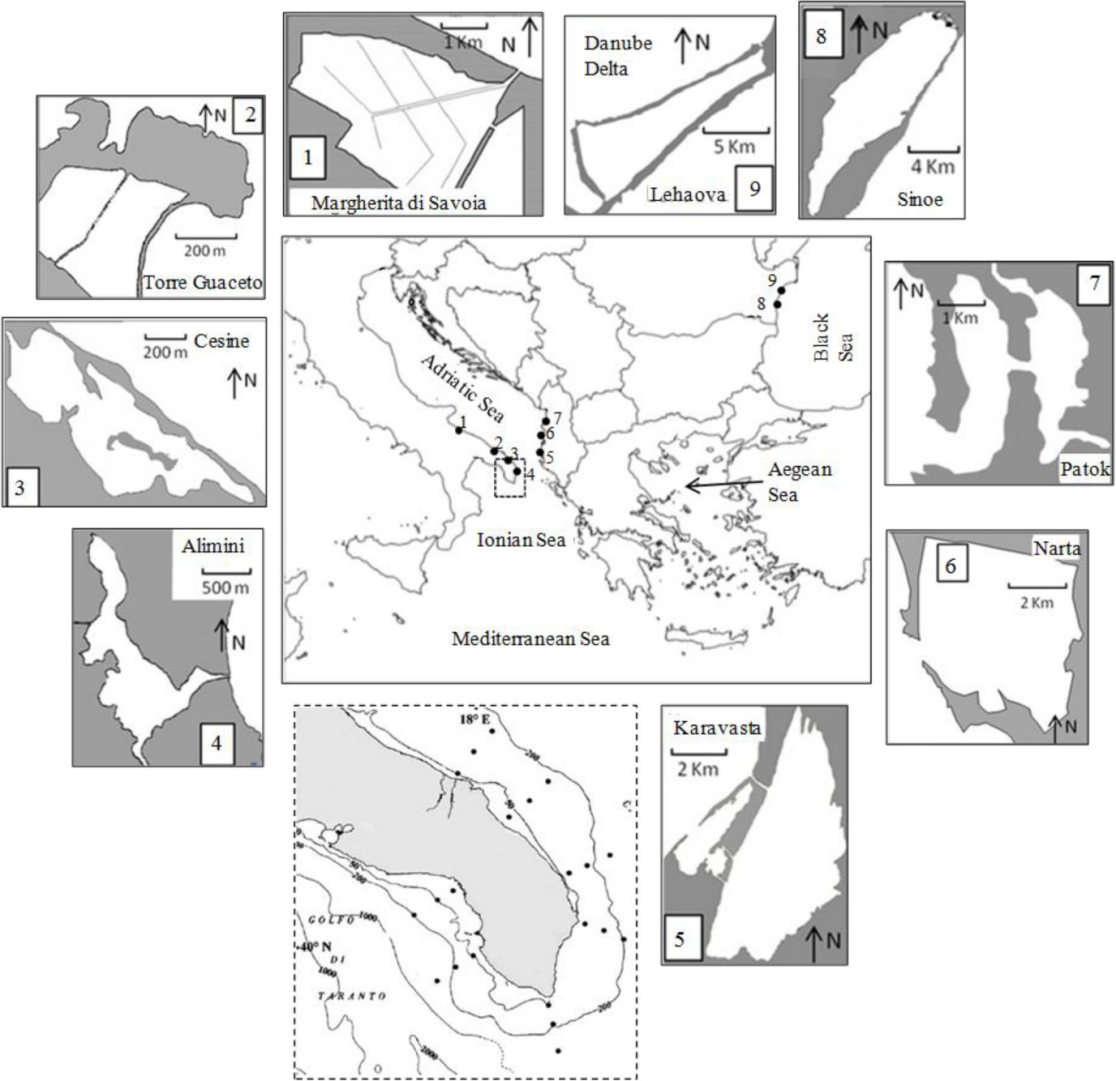
Study sites. From 1 to 9 boxes represent the coastal lagoons in South-Eastern Mediterranean and Black Sea and the dashed box represents the marine coastal waters around the Salento peninsula (Southern Italy).

The field data were collected from marine coastal waters around the Salento peninsula (Southern Italy) and from shallow inland transitional waters in South-Eastern Mediterranean and Black Sea (Fig 1). Coastal water data were collected during four oceanographic cruises carried out in March, June, September and December 2000 along the Southern coast of Puglia (Adriatic and Ionian Seas, SE Italy). The Southern Adriatic/Eastern Ionian area is characterized by oligotrophic conditions and low primary productivity. Nutrient supply to the shelf area strongly depends on inputs of surface waters along the coast (which in the Salento Peninsula are more abundant in the Northern part) as well as on inputs of groundwater [52]. A superficial southward coastal current flowing from the Northern Adriatic basin and a deep north-westward current flowing from the Eastern Mediterranean (Levantine Intermediate Waters, LIW) also contribute to nutrient supply in the shelf area, although their influence on the Southern Adriatic basin is generally limited, on both temporal and spatial scales [53]. Furthermore, nutrient availability in the euphotic zone depends strongly on vertical stratification/mixing processes [38]. Transitional water data were collected from 9 non-tidal shallow lagoons [54] in the South-Eastern Mediterranean and Black Sea. All selected lagoons are (at least partially) inside protected areas, with a degree of protection ranging from Biosphere Natural Reserve to Nature 2000 special protected areas. They include: Margherita di Savoia, a saltpan consisting of a system of shallow ponds, Torre Guaceto, Cesine and Alimini Grande along the Southern Adriatic coast of Italy; Patok and Karavasta and a second saltpan, Narta, located on the coast of Albania; and Sinoe and Leahova on the Romanian seashore in the Southern part of the Danube River Delta (Fig 1).

### Data collection and laboratory methods

The study is based on two types of data: (1) abiotic data (selected environmental variables) and (2) phytoplankton data (biomass as chlorophyll *a* concentration and morphological traits as cell volume and surface to volume ratio based on individual cell size in nano/micro-phytoplankton guilds). The environmental variables considered in the study were selected on the basis of their expected importance to phytoplankton community as niche axes along three main dimensions: resource (dissolved inorganic nitrogen (DIN), soluble reactive phosphorus (SRP) and soluble reactive silicate (SRSi) concentrations, nitrogen to phosphorus ratios (N/P) and chlorophyll *a*, (chl *a*); mixing proxy (water column stability and depth as factors limiting light and nutrient availability); and physical-chemical parameters (water temperature, salinity and oxygen content). Chl *a* concentration was included into the resource dimension because it is often used as a proxy of primary productivity and of resource availability for phytoplankton [55, 56]. In the physico-chemical dimension, temperature was selected due to its expected negative relationship with cell size arising from size-dependent metabolic demand [21, 57], salinity due to its expected positive relationship with cell size arising from osmoregulation costs [58, 59], and oxygen content as a measure of the overall biological equilibrium [60]. A comparison of the selected environmental variables in the coastal and transitional water environments considered in this study is showed in Table 1.

**Table 1:** Physico-chemical characteristics of coastal (CW) and transitional waters (TW).

	CW		TW		
	Mean	2SE	Mean	2SE	*p*
T(°C)	17.43	0.57	15.40	1.25	**
S	38.64	0.02	28.07	5.96	***
DO (mgL^-1^)	6.09	0.11	7.90	0.60	***
DIN (μmol L^-1^)	2.00	0.51	25.74	6.81	***
SRP (μmol L^-1^)	0.14	0.02	1.18	1.51	ns
SRSi (μmol L^-1^)	1.74	0.21	14.06	5.17	***
chl a (μgL^-1^)	0.24	0.03	4.29	1.58	***
N/P	20.05	6.23	277.58	75.72	***
depth (m)	29.79	3.18	0.69	0.16	***
Brunt-Vaisala frequency (s^-1^) (*)	1.24	0.14	-	-	-

T, temperature; S, salinity; DO, dissolved oxygen; DIN, dissolved inorganic nitrogen; SRP, soluble reactive phosphate; SRSi, soluble reactive silicate; chl a, chlorophyll a. SE = standard error representing the environmental variability within coastal and transitional waters; ns = not significant; ** = p<0.01; *** = p<0.001. (§) Index of column water stability as described in Sabetta et al. 2008. Transitional waters are too shallow and too affected by wind conditions to yield useful values for this parameter.

In coastal ecosystems, samples were collected along seven transects running perpendicular to the coastline with three stations per transect at 3, 9, and 15 nautical miles from the shore (Fig 1). For each station and sampling cruise, temperature, salinity and dissolved oxygen were obtained with a SBE 9/11 Plus CTD. Water samples for phytoplankton and nutrient analysis were collected using a Carousel sampler equipped with 12 Niskin bottles. Water samples for phytoplankton analysis were collected at three depths, including the deep chlorophyll maximum (DCM) determined by the fluorescence measurements. Water column stability was determined with reference to the Brunt–Väsälä frequency as described in Sabetta et al. [38]. From the vertical profiles of salinity and temperature, the density variation was calculated and then averaged at 1 m intervals. The vertical density gradient was used to calculate water column stratification as a Brunt—Väsälä frequency according to the equation:

$$N^{2}\left(\text{z}\right)=-\frac{g}{\rho \left(z\right)}\;\frac{d\rho \left(z\right)}{dz}$$

where *g* is gravitational acceleration, *ρ* the density value at *z* depth and *dρ* the density difference over the *dz* depth interval, equal to 1m and as the difference between the densities at the shallowest and deepest sampling points. Given that mean depth in transitional waters is less than 1 m and available data are at surface level we assume that water column stability is much smaller than coastal waters. In transitional ecosystems, physico-chemical data and water samples were collected during autumn 2004 and spring 2005 from the two or three dominant habitat types within each ecosystem. Four replicate samples were collected at a single station from each habitat type, defined according to an intra-habitat classification. Water samples were collected just below the water surface (average depth 0.5 m) using Ruttner bottles. At each station and sampling date, temperature, salinity and dissolved oxygen were measured with a hand-held multi-probe meter (YSI 556). The same analytical procedures were used to analyse nutrient concentrations and phytoplankton community at each site in both ecosystem types. Sub-samples for nutrient analysis were filtered through GF/F filters and stored at -20°C in 100 ml LDPE (low density polyethylene) bottles until analysis. In the laboratory, nitrate, nitrite, ammonium and phosphate concentrations were determined using a Technicon II Auto analyzer, as in Hansen and Grasshoff [61]. For each sampling, an aliquot was filtered directly onto 25 mm GF/F Whatman filters for chlorophyll *a* determination. Filters were stored in a freezer at -20°C until determination. Spectro-fluorimetric analyses of Chl *a* were carried out as in Holm-Hansen [62]. Filters were placed in neutralised 90% v/v acetone and allowed to extract for 2 h. The extract was analysed, before and after acidification, with a Shimazu RF1501 spectrofluorimeter.

### Phytoplankton taxonomic identification and determination of morphological traits

Sub-samples for phytoplankton taxonomic identification and cell size / abundance analysis were preserved with Lugol (15 ml/l of sample). Samples were observed with a Nikon T300E inverted microscope following Utermöhl’s method [63] and identified to species level where possible [64]. In coastal and transitional water sub-samples, linear measurements of 200 and 400 cells, overall for an amount of more than 130,000 data were measured at 400x magnification with a microscope connected to a video-interactive image analysis system (L.U.C.I.A, Version 4.8, Laboratory Imaging s.r.o.). Then, the calculation of volume was based on geometric approximations assigning a geometric shape for determination of surface area (S) and volume (V) [65, 66, 67]. As a general rule, traits were calculated for counting units, most often this means single cells and sometimes 100 μm threads, coenobiums and colonies (e.g. in some filamentous cyanobacteria) where cells are not observable [68]. Phytoplankton size distributions were obtained from individual cell volume (μm^3^), after log-transformation and class width definition (class width = 1).

### Data analysis and statistics

Coastal waters were sampled at 21 stations in four seasons (n = 84 samples) and transitional waters were sampled from three to seven stations for each lagoon in spring and autumn (n = 54 samples). The environmental variability within both coastal and transitional waters is expressed by means the standard error of the physico-chemical parameters sampled at each station during the whole sampling period in both transitional and coastal waters. On the other hand, the environmental variability between coastal and transitional waters was analysed using one-way analysis of variance (ANOVA). Spearman’s rank correlation was used to assess the quantitative relationships between phytoplankton size and S/V and environmental variables considered as niche dimensions. The Spearman’s rank correlation analysis was performed at community level within and between the two typologies of environments. The morphological variability within both coastal and transitional waters is expressed by means the standard deviation of cell volume and S/V at hierarchical class level. The morphological variability between marine and transitional water phytoplankton were analysed by analysis of variance (ANOVA) and Tukey’s Honestly Significant Difference test (HDS) in order to test significant differences (p<0.05). Before analysis, environmental and morphological data were log (x + 1) transformed to satisfy the assumption of normality and homogeneity of variance. We also used the first and fourth quartiles (25th and 75th percentiles) of the log volume and S/V as summary statistics to characterize the whole trait distribution of phytoplankton community for both coastal and transitional waters in order to highlight differences in cell morphology between the two environments (ANOVA test). Analyses were performed using the STATISTICA software package (Version 7). The relationships between phytoplankton size structure (all environments pooled) and the environmental variables were evaluated by multivariate ordination Canonical Correspondence Analysis (CCA) following Ter Braak [69]. The statistical significance of the relationship between the environmental variables and phytoplankton size classes was assessed using the Monte Carlo permutation test, performing 999 permutations per test. Before analysis, biological and environmental data were log (x + 1) to satisfy the assumption of normality-distributed data. Analysis was performed using the CANOCO software 4.5 package.

## Results

Because the main goal was to test differences in size and surface to volume ratio of phytoplankton between coastal and transitional waters, the results will be presented to highlight these differences comparing the two different environments.

### Environmental parameters

The coastal and transitional water ecosystems sampled in this study showed a wide range of physical-chemical and morphological characteristics that reflected a broad range of environmental factors that affect phytoplankton cell size. All variables showed different values, respectively, on average from coastal to transitional waters in salinity (from 38.64 to 28.07), in nutrient concentrations (DIN from 2 to 25.74, SRP from 0.14 to 1.18 and SRSi from 1.74 to 14.06 μmol L^-1^), in chlorophyll *a* concentration (from 0.24 to 4.29 μmol L^-1^) and in column water depth (from 29.79 to 0.69 m). All variables, including N/P ratio and water column stability are significantly different between transitional and coastal waters, excepted SRP (Table 1; ANOVA test). Transitional waters had higher nutrient and chlorophyll *a* content and higher N/P ratio than coastal waters, as well as being shallower and more fully mixed. Moreover, all parameters considered in this work, were more variable in transitional than coastal waters considering the standard error as descriptor of environmental variability (Table 1). The average SRP value in transitional waters is strongly dependent on two exceptionally high values observed at a single highly hyper-haline site (F ratio test, p<0.01).

### Phytoplankton taxonomic composition and abundance

**Fig 2:**
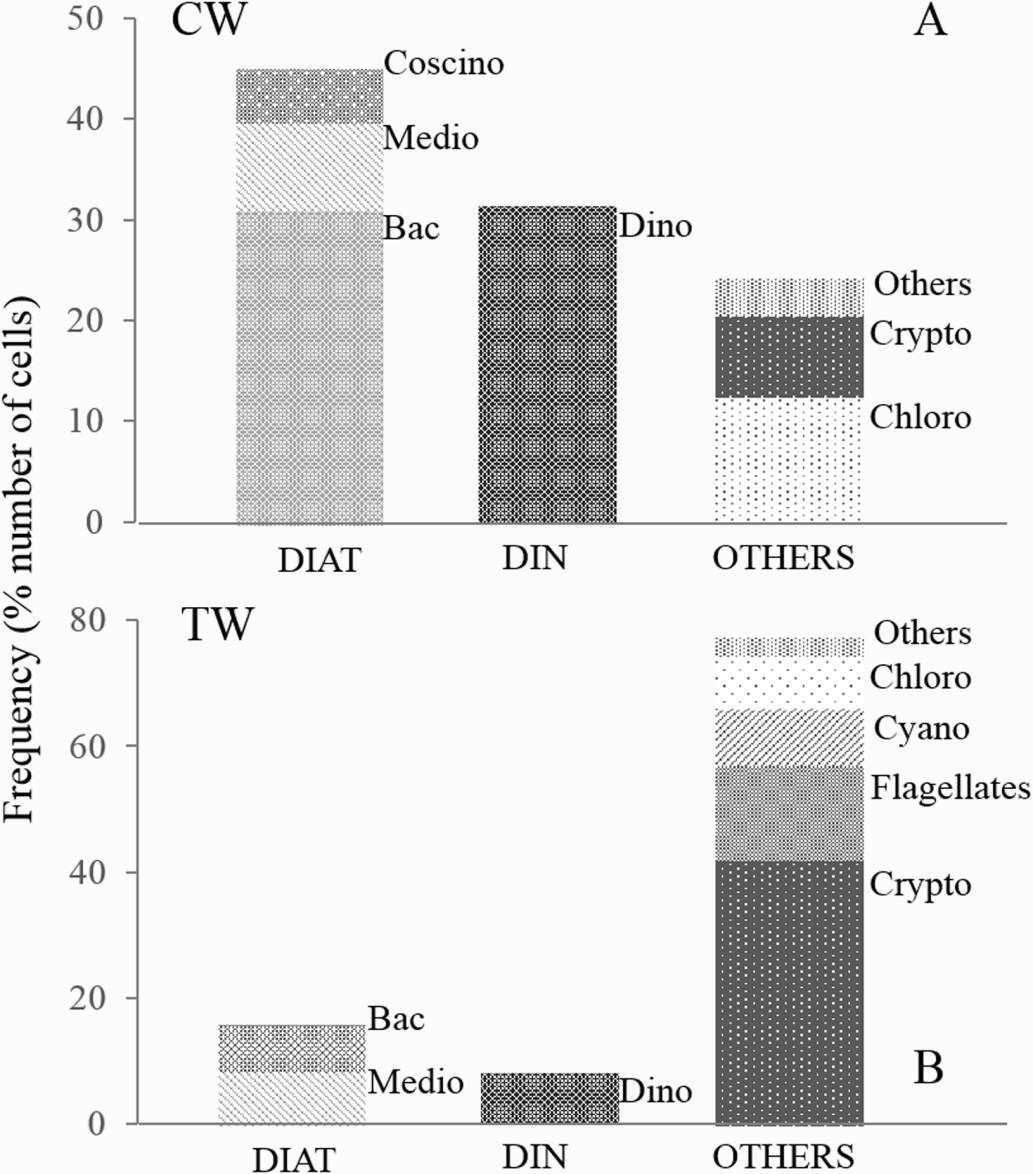
Phytoplankton taxonomic composition in marine and transitional waters. (A) Numerical abundance (number of cells) of phytoplankton community in marine coastal waters and (B) in transitional waters. DIAT = Diatoms (Bac = Bacillariophyceae, Medio = Mediophyceae, Coscino = Coscinodiscophyceae); DIN = Dinoflagellates (Dinophyceae); OTHERS = all other classes (here we report classes that reach higher abundance: Crypto = Cryptophycea, Chloro = Chlorophyceae, Cyano = Cyanophyceae, Flagellates = small undetermined flagellates, Others = all other classes accounting for low percentage).

Overall, 386 taxa were identified during the study, 182 of which were exclusively found in marine coastal waters (CW), 123 only in transitional waters (TW), and 81 in both environments. In coastal waters, species belonging to diatoms (Bacillariophyceae, Mediophyceae and Coscinodiscophyceae) and dinoflagellates (Dinophyceae) were dominant in terms of both cell numerical abundance, accounting for 44.52% and 31.35% respect the overall phytoplankton abundance, and species richness, accounting for 42.59% and 41.06% respectively of identified taxa. Others, consisting of ten phytoplankton classes and other undetermined phytoplankton, collectively accounted for 24.13% of cell abundance (Fig 2A) (see Table 2 for a complete list of phytoplankton classes in CW and TW). In terms of abundance, Bacillariophyceae and Dinophyceae reached on average 8.73x10^6^ and 6.77x10^6^ cell/L, respectively. In transitional waters, the most representative phytoplankton belonging to classes of Other group where Cryptophyceae that accounted for 41.28% of sampled cells, Chlorophyceae for 8.28%, Cyanophyceae for 8.70% and other undetermined phytoplankton accounted for 14.91% of identified taxa. Diatoms and dinoflagellates accounted for only 15.60% and the 8.17% of cell abundance (Fig 2B) and 25.98% and 15.20% of taxa richness, respectively. In terms of abundance, classes belonging to Others reached higher abundance with on average 2.18x10^8^, 1.72x10^8^, 1.40x10^8^ cells/L for Chlorophyceae, Cyanophyceae and Cryptophyceae, respectively. At the species level, six phytoplankton common species were identified that contributed 99% of cumulative abundance in both ecosystems. Four of these, *Chaetoceros wighamii* Brightwell 1856, *Ceratoneis closterium* Ehrenberg 1839, *Navicula transitans* Cleve 1883 and *Licmophora flabellata* (Grev.) C. Agardh 1831, are diatoms and two of these, *Prorocentrum micans* Ehrenberg 1834 and *Scripsiella trochoidea (Stein)* Balech ex Loeblich III 1965 are dinoflagellates.

**Table 2:** Phytoplankton morphological traits.

		CW					TW								
	Class	n ind	V	sd	S/V	sd	n ind	V	sd	S/V	sd		*p*(V)		*p*(S/V)
Diatoms															
	Bacillariophyceae	14263	4684.88	236882.65	2.42	1.97	6367	1090.55	5553.33	1.04	0.51	←	***	←	***
	Coscinodiscophyceae	2460	26190.98	130367.58	0.47	0.22	44	11334.22	42941.12	0.86	0.45	←	***	→	
	Mediophyceae	3968	4748.15	19185.57	0.72	0.48	7317	340.98	791.35	1.17	0.37	←	***	→	***
Dinoflagellates															
	Dinophyceae	14568	10813.78	115299.36	0.62	0.43	7191	4449.67	8604.29	0.63	0.43	←	***	→	***
Others															
	Chlorophyceae	139	18848.30	84134.83	0.63	0.19	7290	249.68	1996.45	2.05	1.25	←	***	→	***
	Chrysophyceae						111	71.28	96.49	1.38	0.27				
	Cryptophyceae	3702	102.69	178.91	2.09	0.82	36322	160.98	186.09	2.88	0.26	→	ns	←	***
	Cyanophyceae	5	607713.70	910360.74	0.33	0.38	7658	329.18	1849.41	1.95	1.05	←	***	→	***
	Dictyochophyceae	585	23271.96	58814.50	0.27	0.11	1	116.60	-	1.59	-	←	-	→	-
	Ebriophyceae	21	1136.77	945.36	0.52	0.12									
	Euglenophyceae	35	18583.18	20820.78	0.31	0.12	70	352.66	697.68	1.36	0.56	←	***	→	***
	Haptophyta incertae sedis	102	13524.88	15037.07	0.25	0.08									
	Prasinophyceae	1	5375.19	-	0.28	-	315	142.54	157.41	1.91	1.22	←	-	→	-
	Prymnesiophyceae	804	17330.04	55348.37	0.35	0.20	47	10613.17	14562.05	0.80	0.53	←	ns	→	***
	Trebouxiophyceae	1	2726.16	-	4.27	-	112	46.70	41.59	1.87	0.43	←	-	←	-
	Ulvophyceae						1606	82.93	92.04	1.85	0.61				
	Zygnematophyceae						74	455.83	296.57	3.32	0.91				

n ind = number of individuals; cell size = mean cell volume; sd = standard deviation; S/V = surface to volume ratio. ANOVA results applied to water categories. ns = not significant; *** = p<0.001. Arrows indicate increasing cell size and S/V values.

### Morphological traits: volume and surface to volume ratio

**Fig 3:**
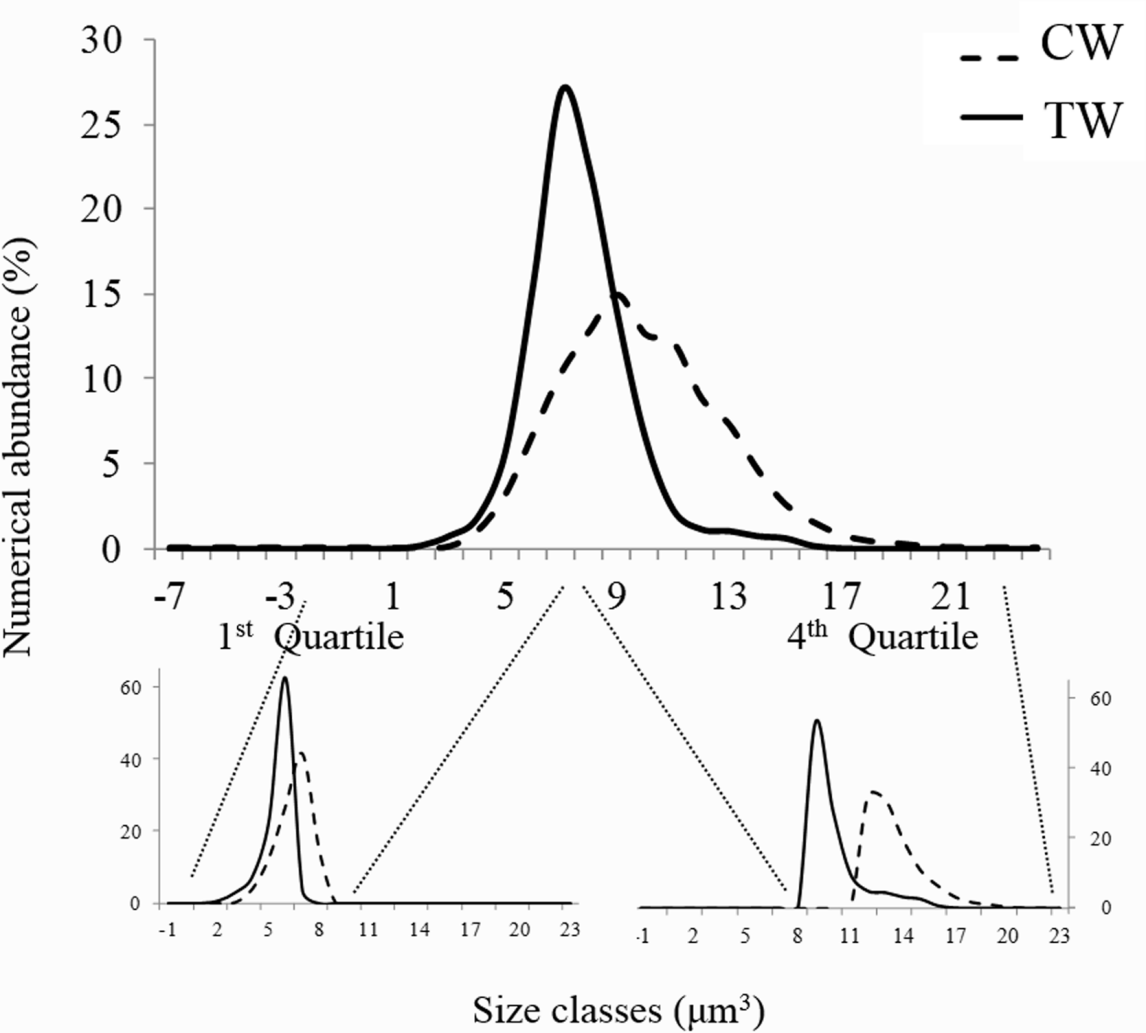
Size abundance distribution of nano-microphytoplankton. (A) in marine coastal (CW) and transitional waters (TW) across the full size spectra (A); in the first quartile (B) and in the fourth quartile (C) of the phytoplankton log volume and surface to volume ratio.

**Fig 4:**
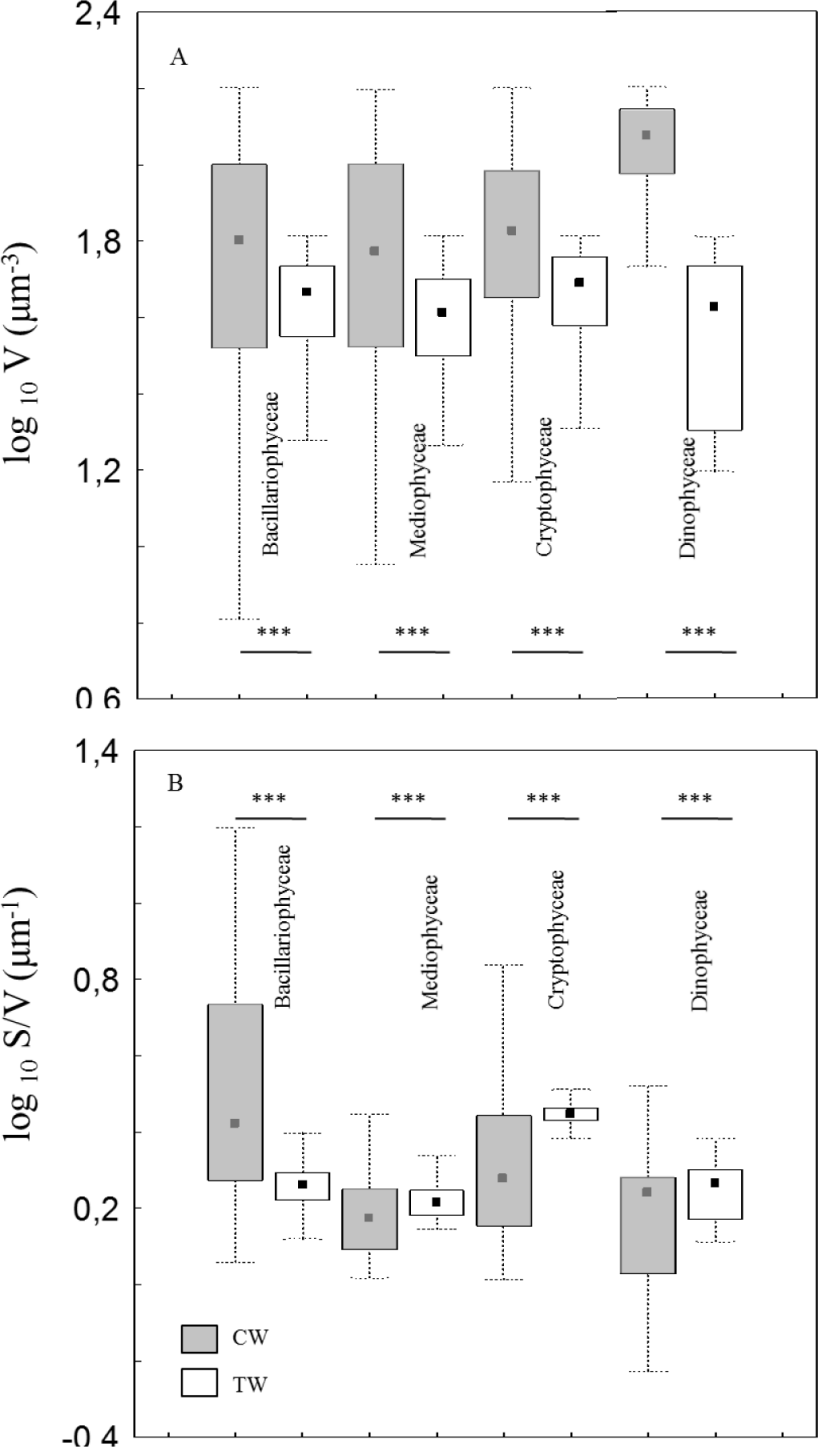
Small phytoplankton traits distribution. Box-Whisker plots of cell size (A) and S/V (B) of common phytoplankton classes in CW and TW environments of first quartile. Data are arranged in order of increasing medians in CW. Pairwise comparisons were run on log-transformed data (ns = not significant; *** = p<0.001).

**Fig 5:**
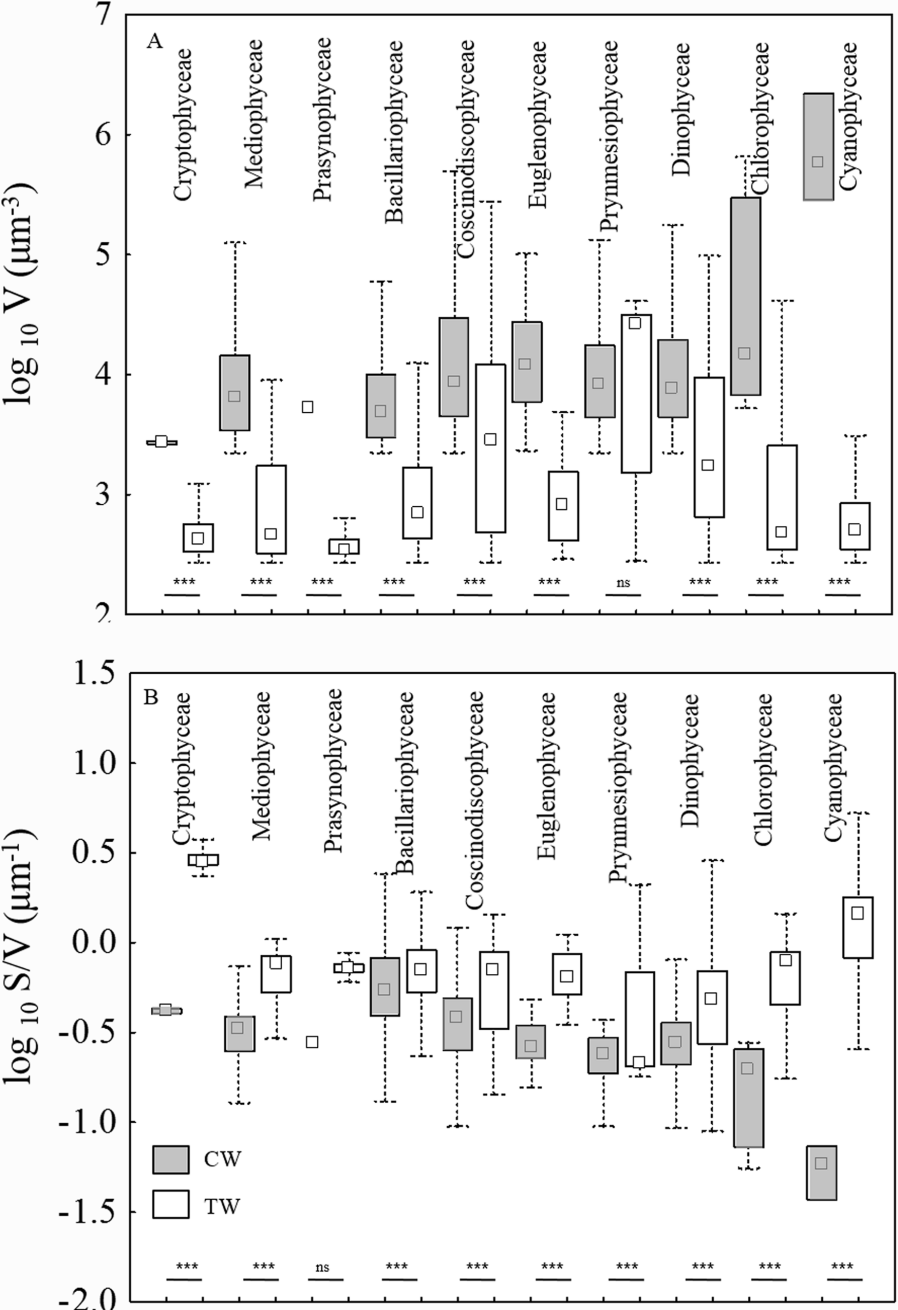
Large phytoplankton traits distribution. Box-Whisker plots of cell size (A) and S/V (B) of common phytoplankton classes in CW and TW environments of fourth quartile. Data are arranged in order of increasing medians in CW. Pairwise comparisons were run on log-transformed data (ns = not significant; *** = p<0.001).

Coastal phytoplankton covered a wider range of size, spanning 6 orders of magnitude in cell volume from 0.2x10^1^ and 2.7x10^7^ μm^3^, whereas transitional waters phytoplankton vary 4 orders of magnitude ranging from 0.05x10^1^ and 2.7x10^5^. Mean cell volume of marine phytoplankton was two orders of magnitude greater than in transitional waters (7.6x10^3^ μm^3^ ± 1.5x10^5^ *vs*. 0.6x10^3^μm^3^± 3.4x10^3^) significantly difference (ANOVA test: F_1,134471_ = 187.8, p<0.001). The whole community size distribution, which reflect the overall phytoplankton size abundance relationships, were hump-shaped in both environments, with a shift in the distribution mode towards larger size in coastal than transitional waters (Fig 3A). The same shift was observed in both first and fourth quartile (25th and 75th percentiles) of the whole size distribution (Fig 3B and 3C). Mean phytoplankton cell volume both in the first and the fourth quartile was larger in coastal than in transitional waters (ANOVA test: F_1,33615_ = 6339.1, p<0.001 and F_1,33618_ = 86.13, p<0.001, respectively). Significant differences were observed in phytoplankton cell volume in both coastal and transitional waters at class and species taxonomic level. Phytoplankton classes are listed in Table 2. In total, 12 phytoplankton classes are common to the coastal and transitional water datasets; mean cell volume was larger in coastal than in transitional waters in 11 out of 12 classes, and significantly larger in the nine classes for which a statistical comparison was possible (Table 2). Concerning the second morphological trait considered here, i.e., the surface-to-volume ratio of individual cells (S/V), was significantly lower in coastal than transitional waters in 7 out of the 9 classes for which a statistical comparison was possible (Table 2). When morphological traits were observed within first and fourth quartile of size distribution, similar patterns were found. In the first quartile, for all four common classes, (Bacillariophyceae, Mediophyceae, Cryptophyceae and Dinophyceae) mean cell volumes were significant larger in coastal than transitional waters (Fig 4A) and the surface to volume ratio was smaller in coastal than in transitional waters with the exception of Bacillariophyceae (Fig 4B). In the fourth quartile, 10 classes were found in both coastal and transitional waters (Fig 5). Mean cell volume was larger in coastal than in transitional waters in every class with the exception of Prymnesiophyceae (Fig 5B); the surface to volume ratio was smaller in coastal than in transitional waters in every class (Fig 5C).

**Fig 6:**
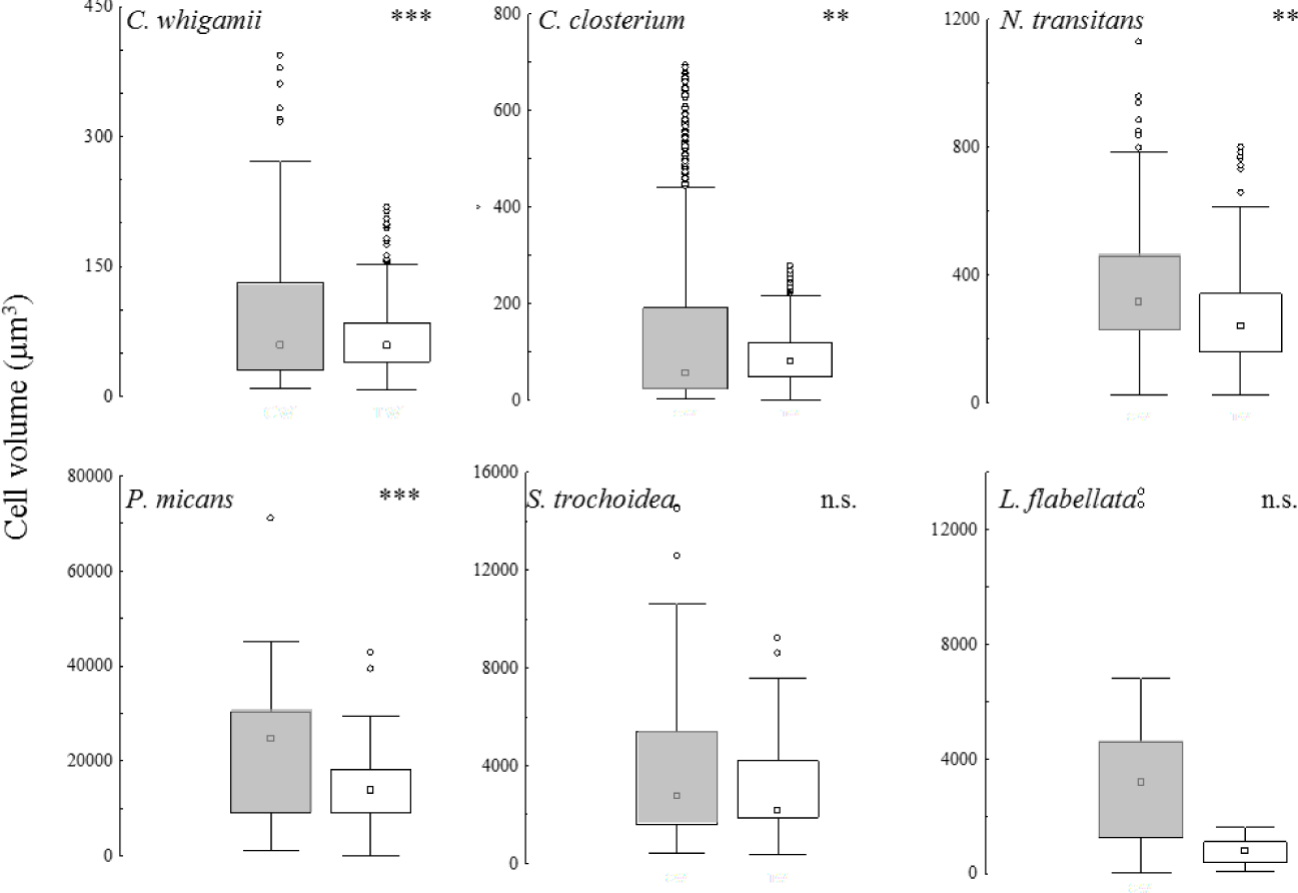
Differences in size of phytoplankton at species hierarchical level. Box-Whisker plots of cell size of species common to CW and TW that contributed 99% of cumulative abundance in both environments. Pairwise comparisons were run on log-transformed data (**p = <0.01, *** = p<0.001).

At the species level, the six species that contributed 99% of cumulative abundance in both environments also had significant larger cell volume in coastal than transitional waters. Cell volume was significantly larger in coastal than transitional waters with exception for *S*. *trochoidea* and *L*. *flabellata*, although they showed the same trend (Fig 6). In each case, marine species distributions showed larger range, means and upper values compared with the transitional waters.

### Abiotic drivers of morphological trait variation

**Fig 7:**
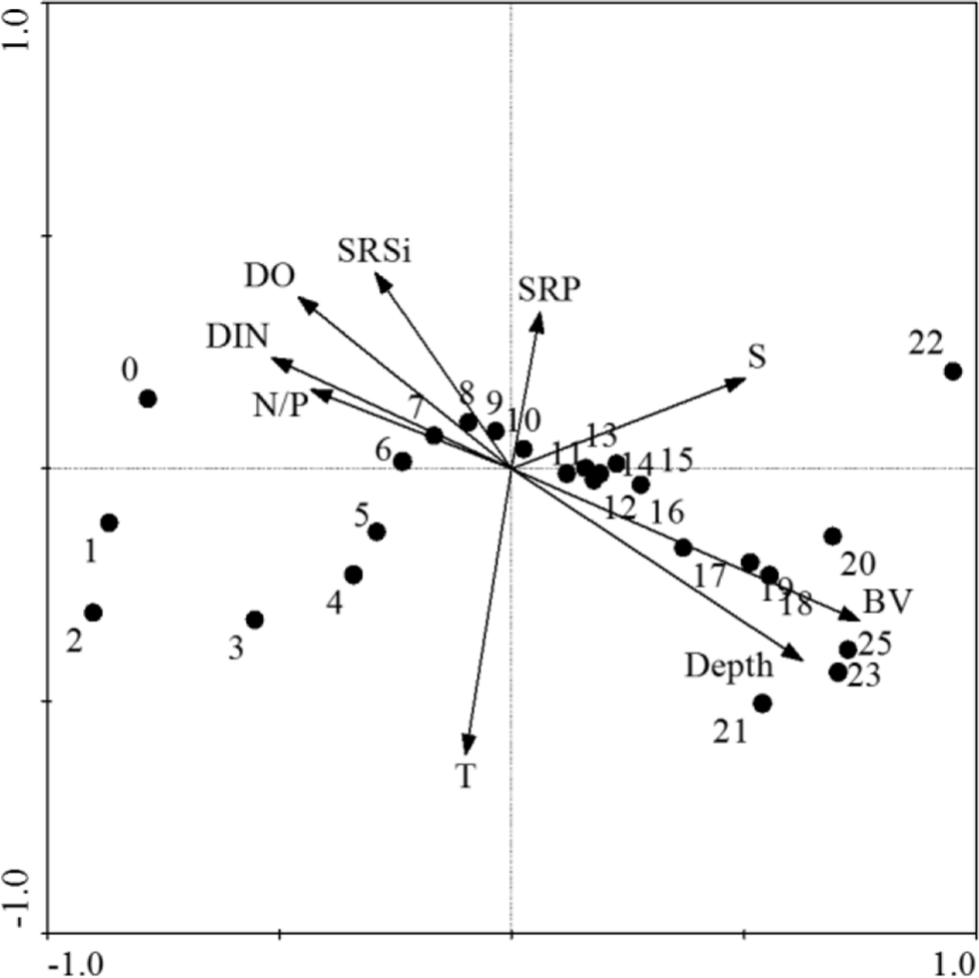
Size classes-environmental variables CCA biplot. Arrows represent the environmental variables; symbols correspond to the size classes. Environmental variables are coded as follows: T (temperature, °C), S (salinity), DO (dissolved oxygen, mgL^-1^), DIN (dissolved inorganic nitrogen, μmol L^-1^), SRSi (soluble reactive silica, μmol L^-1^), SRP (soluble reactive phosphorus, μmol L^-1^), N/P (nitrogen to phosphorus ratio), Depth (m), BV (Brunt-Vaisala frequency, s^-1^). Cumulative percentage variance is 35%.

Canonical Correspondence Analysis (CCA) was conducted to identify patterns of the whole phytoplankton size distribution with respect to pre-selected environmental variables (Fig 7). The length of the environmental variable arrows in the ordination diagram represents the relative importance of each variable in relation to size classes. The CCA analysis revealed that mixing proxy niche dimension (represented by water column stability and depth) and trophic resources niche dimension (represented by DIN, SRSi, and N/P) made significant contributions (P<0.05) to the variance, providing a good representation of the major environmental factors controlling phytoplankton size structure. The eigenvalues of the first two canonical axes (0.57 and 0.14, respectively) explained 30% of the total variance. The CCA ordination highlighted a clear pattern: the position of the phytoplankton size classes varied along the horizontal axis and it was mainly determined by a gradient based on nutrient concentration and water column depth and stability. Large phytoplankton size classes, located in the right-hand quadrants show a positive relationship with water column depth and stability whereas small phytoplankton size classes, mostly located in the left-hand quadrants are positively associated with the increasing concentration of nutrients (Fig 7). Table 3 shows correlation matrix of environmental variables, morphological traits (size and surface-to-volume ratio) and taxonomical aggregation (pooled and classes), for TW, CW and both environments combined. Across the two environments and all classes, phytoplankton cell size was found to be positively related to water column stability components (Table 3) and negatively related to all trophic resource components other than SRP and SRSi (Table 3). Considering coastal and transitional waters separately, in the former, phytoplankton cell size was negatively related to SRP and positively related to N/P; diatoms cell size increased with DIN. In the latter, even though not significantly, phytoplankton cell size was positively related to SRP concentration (Table 3). Across the two environments, the phytoplankton S/V ratio decreased with water column stability and depth while increased with DIN, chl*a* and N/P ratio in the guild as a whole and in others group. Diatoms showed an opposite pattern, its S/V ratio positively correlated with water column depth and stability and negatively correlated with DIN, chl*a* and N/P ratio (Table 3). Within each environment, S/V ratio in coastal waters was inversely related to water column stability considering the whole phytoplankton community. The main taxonomic groups showed contrasting patterns along the trophic resource dimension: S/V was negatively related to DIN in diatoms and dinoflagellates and positively related to SRP in all groups (Table 3). In transitional waters, phytoplankton cell S/V ratio does not show significant relationships to abiotic niche dimension (Table 3).

**Table 3:** Niche dimensions and phytoplankton morphological traits.

		Mixing proxy dimension		Trophic resources dimension					Physico—chemical dimension		
Size (um^-3^)		Depth	BV(α)	DIN	SRP	SRSi	chla	N/P	T	S	DO
CW—TW											
	DIAT	**0.46**	**0.53**	**-0.39**	0.04	-0.02	**-0.43**	**-0.35**	-0.05	0.11	**-0.31**
	DIN	**0.35**	**0.38**	**-0.28**	-0.07	0.04	**-0.34**	**-0.22**	-0.15	0.14	**-0.18**
	OTHER	**0.61**	**0.71**	**-0.59**	-0.02	**-0.19**	**-0.58**	**-0.50**	-0.03	**0.24**	**-0.34**
	TOTAL	**0.62**	**0.72**	**-0.38**	-0.02	-0.14	**-0.37**	**-0.49**	0.03	**0.22**	**-0.14**
CW											
	DIAT	-0.21	-0.01	**0.24**	-0.09	0.13	0.17	**0.27**	-0.18	0.06	0.02
	DIN	-0.12	-0.07	0.20	**-0.23**	0.09	0.22	**0.32**	-0.19	0.12	0.12
	OTHER	-0.11	0.16	0.09	-0.21	0.02	0.09	**0.26**	-0.16	0.14	**0.26**
	TOTAL	-0.17	0.13	0.13	**-0.27**	0.02	0.11	**0.30**	-0.12	0.15	**0.23**
TW											
	DIAT	-0.04	-	0.08	0.05	0.21	**-0.40**	0.06	-0.17	-0.01	0.06
	DIN	-0.13	-	0.15	0.15	-0.26	0.27	0.23	**0.36**	0.11	-0.23
	OTHER	0.25	-	0.10	0.10	-0.04	-0.16	0.01	-0.03	0.03	0.03
	TOTAL	-0.01	-	0.01	0.19	0.23	-0.24	0.10	-0.20	-0.08	-0.13
S/V (μm^-1^)											
CW—TW											
	DIAT	**0.46**	**0.43**	**-0.45**	**0.19**	**-0.20**	**-0.38**	**-0.49**	**0.22**	**0.20**	**-0.34**
	DIN	-0.05	-0.13	0.02	0.11	-0.14	0.18	-0.05	**0.32**	-0.04	-0.08
	OTHER	**-0.61**	**-0.76**	**0.63**	0.06	**0.19**	**0.68**	**0.53**	-0.12	**-0.26**	**0.37**
	TOTAL	**-0.29**	**-0.46**	**0.37**	0.11	0.07	**0.51**	**0.27**	0.02	**-0.18**	**0.20**
CW											
	DIAT	0.13	0.02	**-0.26**	**0.26**	-0.10	-0.14	**-0.39**	**0.30**	0.08	-0.10
	DIN	0.22	0.05	-0.21	**0.30**	-0.10	-0.16	**-0.40**	**0.28**	**-0.30**	-0.18
	OTHER	0.01	**-0.44**	0.14	**0.30**	0.10	0.09	-0.10	0.06	-0.15	**-0.40**
	TOTAL	0.10	**-0.29**	-0.07	**0.29**	0.11	0.16	**-0.28**	0.12	-0.20	**-0.35**
TW											
	DIAT	0.04	-	-0.01	-0.16	-0.08	-0.16	0.07	0.05	0.09	0.30
	DIN	-0.13	-	0.15	-0.15	-0.26	0.26	0.22	**0.36**	0.12	0.17
	OTHER	0.26	-	0.05	0.10	-0.04	-0.15	0.01	-0.03	-0.22	0.23
	TOTAL	0.02	-	0.03	0.08	-0.19	-0.16	-0.01	0.06	-0.21	0.21

Relationship of environmental variables and cell volume and S/V within and between environments. (α) TWs are too shallow and too affected by wind conditions to yield useful values. Values in bold indicate significant correlation between variables.

## Discussion

We have found significant smaller average cell size and larger surface to volume ratio of phytoplankton with increasing nutrient concentration along sharply different mixing conditions comparing size distributions in coastal marine and shallow transitional waters. Size distributions of phytoplankton communities and their relative first and the fourth quartile showed differences between the two environments with smaller size range, means and upper values in transitional than in coastal waters (Fig 3A–3C). Zooming at taxonomic class level, we have found smaller cell size with higher S/V in transitional than in coastal waters (Figs 4 and 5). At the species level, the common species in both environments had consistently smaller size in transitional water ecosystems than conspecifics from coastal marine ecosystems (Fig 6). The observed shift in cell size does not seem to depend on taxonomic composition, since the differences were consistently observed at every level of resolution, from the whole community to the species level. Moreover, it does not depend on the geographical location of the study sites because the same shifts in size have been observed when comparing phytoplankton communities in different geographical areas (i.e., coastal marine areas and lagoons around the Salento peninsula). Finally, even though the work is based on different sampling efforts, we assume that the time lag does not produce significant bias and the comparison is plausible because the structure of column water is similar in March-December *vs* June-September and the phytoplankton community related to the environmental factors showed similar size pattern in the period March-December *vs* June [38].

Cell size is considered to be an adaptive trait of phytoplankton, with optimal cell size expected to reflect responses to environmental variables [70]. Aquatic environments differing in many physical and chemical characteristics may impose different selection pressures on phytoplankton cell morphology and adaptive strategies [26, 71, 72, 73]. The best evolutionary strategy for phytoplankton is supposed to minimize cell size and maximize surface-to-volume ratio [28, 74] in order to acquire nutrients more effectively and reduce sinking losses [75] bringing the question of what leads to the evolution of large phytoplankton cells [26, 74]. Large-sized cells adopt several behavioural and physiological strategies to survive under high- or/and fluctuating nutrient supply [5, 26, 44], high- and fluctuating light conditions [29, 40, 47], increasing mixing conditions [41] then, why don’t large cells dominate in transitional waters environment?

### Niche dimensions as potential drivers in determining morphological cells adaptation

Assuming that shift in phytoplankton size distributions comparing different environments resulted from directional selective pressures along niche dimensions [26], here, we hypothesize why lagoon conditions do not select for large cells. Our results suggest that water column depth and water column stability conditions likely provide direct effect on cell size across the whole size distribution favouring large cells in deeper and stable conditions and small cells in shallower and frequently and complete mixed conditions (Fig 7). The interplay between mixed layer depth and size-dependent sinking behaviour have already been cited as potential driver of phytoplankton size both for nutrient uptake [26] and light uptake [24].

We found three different reasons to conclude that nutrient supply and concentration as niche dimensions do not fully explain the observed shift in phytoplankton cell size and S/V. First, we obtained contrasting relationships between nutrient concentration and phytoplankton cell morphology when comparing the pooled dataset (transitional vs. marine environments) or within either transitional or coastal waters. On one hand, across environments, phytoplankton cell size decreases with increasing nutrients concentration. On the other hand, within environments cell size increases with increasing concentration of the limiting nutrient, which is assumed to be phosphorus and nitrogen, respectively in transitional and coastal waters. Although phosphorus concentration close to the detection limit are not yet limiting phytoplankton growth [76] and, both nitrogen and phosphorus can limit different species at different time and location in lagoons, overall phosphorus has been considered to be the principal limiting nutrient in transitional environments [77]. Second, we assume that the frequency of nutrient pulses is higher in transitional than in coastal waters due to the short-term processes enhancing nutrient supply [78]. According to empirically based principle, phytoplankton and, particularly, diatoms respond rapidly to episodic high-resources conditions [33, 79, 80]. However, we have found the same shift in size and S/V for all phytoplankton functional groups then, nutrient pulses alone seem not explain the pattern. Third, we focused on the nutrient storage capacity of phytoplankton. The larger cell size of diatoms in coastal marine than in transitional waters can be explained by their nitrogen storage capacity under limiting conditions [81], as already proposed in the comparison between lake and marine environments [26]. On the other hand, in contrast to dinoflagellates and other algae, diatoms have evolved a larger nutrient storage vacuole efficient to retain high concentrations of nitrate and phosphate [82–85]. Moreover, transitional water dinoflagellates and cryptophytes are also smaller than marine ones in spite of their potentially heterotrophic behaviour that might be an additional advantage to increase the size in such eutrophic environments [86].

We assume that the essential property of these shallow water bodies is that much or all of bottom sediment surface is frequently, if not continuously, mixed [87]. However, shallow waters (< 5m) have a theoretical light-supportive capacity [29] with a low extinction coefficient promoting the penetration of solar irradiance to the bottom [88] and providing plenty of scope for the intervention of other potential limiting factors for phytoplankton growth [29]. Photophysiological and behavioural strategies suggest being large is often beneficial in high-light environments [29, 89]. Shallow-mixed layers can induce high- or fluctuating-light conditions by which cells can suffer cellular damage and inhibited rates of photosynthesis and growth due to high light and increased UV penetration [24]. On one hand, larger cells suffer less photo-inhibition than smaller cells [40] and have lower metabolic costs of photosynthetic regulation to endure short-term exposure to high light [47, 90]. On the other hand, large phytoplankton adopt behavioural strategies of depth-regulating to different regimes of turbulence migrating downwards from high irradiance level [29, 91, 92]. The conditions of frequently and complete mixing of the water bodies of this study support the hypothesis that large-sized should have competitive advantage over the smaller-sized phytoplankton. Our results highlight that also the response of cell morphology to light availability as a niche dimension does not match expectations. However, the observed shifts in size distribution might also be determined by differences between transitional and coastal waters in terms of size-selective grazing pressures. There is evidence that in shallow environments grazing pressure depends more on non-selective benthic filter feeders than zooplankton grazers [93–96] in these conditions large cells may adopt buoyancy and swimming strategies in order to suppress sinking in shallow waters [97].

### Understanding mechanisms

We suggest that the size-dependent indirect effect of shallow and well-mixed environment is due to the thinning of the diffusive boundary layer surrounding a cell, which in turn limits nutrient transport from external environment to cell surface. At cellular scale, the re-supply rate of nutrients to the boundary layer is dependent on the nutrient gradient across the boundary layer from the cell surface [41, 98]. Large cells, with their smaller surface to volume ratio than small cells are more likely diffusion-limited. Therefore, larger cells increase gradients and nutrient fluxes by swimming or sinking [98–101]. Theoretically, when nutrient concentrations and turbulent mixing are low, small cells have an adaptive advantage over larger cells because they are more efficient in acquiring limiting nutrients [6, 41]. Nevertheless, our results allow us to hypothesize that size-dependent sinking behaviour does not confer selective advantages in shallow and fully mixed environments. This interpretation rests on the assumption that there is a fitness advantage to large cell size with lower S/V in marine coastal waters, which we have not quantified here.

## Conclusions

We hypothesize that the systematic shift in cell morphological traits observed at various levels of taxonomic resolution and across the full size distribution suggests that the influence of key phytoplankton niche dimensions, such as nutrient availability and light limitation, is in fact limited in this case. The selective strategy of large species arising from sinking behaviour on one hand, ensures resource exploitation in deeper and well-structured waters on the other hand, seem not confer a competitive advantage over small cells in lagoon conditions. Why is the large cell fitness limited in transitional waters? Large phytoplankton have a lower fitness under lagoon than marine environments whereas they are able to explore larger patches with low resources concentration. On the other hand, small cells fit better transitional environments exploring small patches with high resources concentration as already proposed [102, 103]. The competitive advantage in resource exploitation due to the cell size-dependent sinking behaviour and how resources are patchy in space could be proposed and investigated as a plausible mechanism promoting species coexistence and shaping community size structure in marine *vs* transitional water phytoplankton.

## Supporting Information

S1 File Taxonomic list of phytoplankton community in marine coastal and transitional water environments. (XLS)
